# Racial discrimination within United Nations offices in Geneva: Results from an online survey

**DOI:** 10.1371/journal.pone.0295715

**Published:** 2024-01-17

**Authors:** Hannah Strohmeier, Ronald Musizvingoza, Nisha Sajnani

**Affiliations:** 1 Institute of International Health, Charité –Universitätsmedizin Berlin, Berlin, Germany; 2 Steinhart School of Culture, Education and Human Development, New York University, New York, New York, United States of America; Dayeh University, TAIWAN

## Abstract

Racial discrimination adversely impacts health and well-being, and interferes with organizational functioning, including United Nations offices where limited systematic research exists. This article presents and discusses a secondary analysis of data from the ‘Survey on Racial Discrimination’ issued by the United Nations Staff Union Geneva in 2020. The survey produced quantitative and qualitative data and was completed by 1251 staff, consultants, and interns (response rate: 14.7%). Descriptive statistics were computed for key findings. More than one third (34.4%) of participants reported having personally experienced racial discrimination. Most reported national origin as basis (61.8%), stated that this experience had affected their opportunities for career advancement (66.2%), and took no action as response (57.4%), mainly due to a lack of trust in the organization’s recourse mechanism. In addition, more than one third (34.3%) of survey participants had witnessed colleagues being racially discriminated against. Chi-square tests to assess differences between groups showed that those belonging to a racial, ethnic, and/or national minority or group reported higher rates of personally experienced and witnessed incidents of racial discrimination compared to those who did not identify as such. Furthermore, participants who reported having experienced racial discrimination had a higher proportion of witnessing racial discrimination. The qualitative survey data on suggested measures to address racial discrimination in the workplace were examined through thematic analysis and rendered three overarching themes: Understanding racial discrimination; revising practices of recruitment, promotion, and appointment; and restructuring case management processes. Our results suggest that racial discrimination poses a significant issue within United Nations offices in Geneva and call for educational initiatives and significant structural changes. We recommend tailored research to inform these measures and highlight that committed leadership and the participation and vigilance of all involved in shaping the culture of the organization is needed to address racial discrimination in the workplace.

## Background

Racial discrimination (RD) is defined by the International Convention on the Elimination of All Forms of Racial Discrimination (ICERD) in Article 1 as “any distinction, exclusion, restriction or preference based on race, colour, descent, or national or ethnic origin which has the purpose or effect of nullifying or impairing the recognition, enjoyment or exercise, on an equal footing, of human rights and fundamental freedoms in the political, economic, social, cultural or any other field of public life” [1]. Despite strides that have been made to combat RD in the workplace, it remains prevalent in many organizations across the globe [2]. Addressing RD calls for urgent action for several pressing reasons: First, while the experience of RD might differ between individuals, its impacts are oftentimes manifold and at times severe. For example, RD is well known to adversely affect the health of those experiencing it. As Schouler-Ocak and Moran [3] concluded in their recent review on RD and mental health, “a growing body of literature shows that racism is significantly related to poor health, with the relationship being particularly strong for mental health and less robust for physical health.” Second, with regards to the workplace, racial harassment and discrimination have been linked with a higher prevalence of work-related illness, injury, and assault amongst racialized employees as compared to those who identify as white [4–6]. RD, in concert with other forms of discrimination based on gender identity, ability, sexual orientation, and age, for example, have been demonstrated to prompt chronic feelings of exclusion, isolation, and reduced psychological safety which, understandably, have an impact on employee motivation, mental health, and employee relations [7,8]. Workplace discrimination has also been associated with an elevated risk of long-term sickness absence [9]. Compromised employee health and poor work climate can, in turn, also impact organizational functioning [10] and productivity. For example, LaVeist, Gaskin [11] estimated a loss of more than $1 trillion due to illness and premature death related to racial health inequalities in the United States between 2003 and 2006 alone. Finally, according to international law, RD is illegal, with the ICERD being “the centerpiece of the international regime for the protection and enforcement of the right against racial discrimination” [12]. Organizations typically have respective frameworks in place; in the specific context of the United Nations (UN) these include the Charter and the Staff Regulations and Rules, which prohibit RD and present a legal framework for the obligations and rights of UN employees [13].

Despite the above-mentioned adverse effects and the presence of protective legal frameworks, RD—and racism more broadly—pose a significant problem across development and humanitarian organizations including those belonging to the UN system, which stakeholders (practitioners, donors, and academics) now widely acknowledge [e.g., 14–20]. While scholarly research on this issue remains up to this date limited [14,16], existing data and information stem largely from personal accounts released in recent years [e.g., 21–23], or were produced by organizations themselves. For example, in 2019, UNICEF–one of the UN’s Funds and Programs–released the Report of the Independent Task Force on Workplace Gender Discrimination, Sexual Harassment, Harassment and Abuse of Authority [24]. Although the focus of this Task Force was not on RD per se, its report states that “(s)taff also referred to discrimination based on race or ethnicity, particularly in field offices”, and notes that the divide between nationally and internationally recruited staff “seems to manifest in various ways, sometimes as bullying or outright abuse, clear racial discrimination, gender-based discrimination, sexual harassment or cultural insensitivity” [24]. Furthermore, in the wake of the deaths of George Floyd, Breonna Tyler and others in the United States, and the re-emergence of the Black Lives Matter movement, the UN Secretariat in New York launched a survey in late 2020, with the objective “to assess staff perceptions on the extent of racism and racial discrimination in the Organization” [13]. The results of this survey, publicly available as a summary, were based on the responses of more than 8000 Secretariat staff (22% of total staff) and are unambiguous:

“One in three respondents mentioned having experienced discrimination; 49 per cent of those who experienced such discrimination said that they had experienced it occasionally, while 21 per cent reported experiencing it frequently. The most reported forms of discrimination were based on national origin (49 per cent), racial identity (38 per cent) and colour (31 per cent). Of those who did not report an incident of racial discrimination, 72 per cent took no action because they thought that it would yield no outcome, lacked trust, or feared retaliation. Of those who reported an incident of racial discrimination, 52 per cent said that they were dissatisfied or very dissatisfied with the way in which the situation was handled, and only 13 per cent said that they found some form of support or protection against retaliation” [13].

In addition, the analysis of almost 84,000 comments collected as part of this survey “showed that the most negative comments pertained to career progression, senior leadership, and processes” [13]. According to this report, other UN surveys came to similar conclusions [13] but were not published or made publicly accessible.

In this article, we present and discuss the findings from one of these other surveys, specifically the survey on RD undertaken with UN staff in Geneva. A partial summary of the results of this survey are accessible online [25,26]. However, a comprehensive quantitative and qualitative analysis of the entire dataset has not been published. Our findings expand the evidence base of RD within the UN, which has received limited systematic attention, and may further inform in-house anti-racism strategies and plans. Our results also make a valuable contribution to scholarship pertaining to RD in the workplace with a focus on development and humanitarian organizations.

## Methods

### Survey structure and participation

A survey composed of 44 questions pertaining to RD in the workplace was designed by members of the UN Staff Union Geneva. This included questions about the demographics of participants, such as gender, employment grade, years employed with the UN, and belonging to minorities or groups; and questions focused on the personal experience and/or witnessing of RD in the workplace, the ways in which discrimination manifested, and actions taken in response. The survey also asked participants how comfortable they felt discussing issues of RD in the workplace. Most questions were closed-ended, i.e., participants had to select one or more predefined answer options, although choosing the option ‘Other’ allowed participants to elaborate on their particular situation with their own words. Towards the end, the survey also included an open-ended question asking participants what measures they think the UN should take to address RD in the workplace.

The survey was launched in July 2020 through Survey Monkey, and accessible in the English and French languages for three weeks. Eligible for participation were approximately 8500 staff, consultants, and interns working for the United Nations in Geneva, (i.e., the United Nations Office at Geneva [UNOG], which belongs to the UN Secretariat; UN Funds & Programs; and UN Specialized Agencies) [27]. The invitation to take part was circulated via a broadcasting message that reached the UN workforce by email. Participation in the survey was voluntary and anonymous.

As a partial summary of findings were made publicly available [25,26], this study comprises a secondary analysis of data. The first author (HS) received the raw survey data from the UN Staff Union Geneva on 4 May 2023. Written permission to analyze and report the findings was granted by the UN Staff Union Geneva on 6 May 2023. Following this, one collaborating author (NS) sought approval from the New York University (NYU) Institutional Review Board, Human Research Protection Program, who administratively reviewed the referenced study and determined that it did not meet the criteria for NYU’s engagement in research involving human subjects, as it involves a secondary analysis of data (IRB-FY2023-7923). Since the data were collected by the UN Staff Union Geneva, the authors did not obtain informed consent. None of the authors had access to information that could directly identify individual participants during or after data collection.

### Data analysis

The survey produced quantitative and qualitative data. We used Stata 16 for the quantitative analysis and created dummy variables for having experienced RD; having witnessed RD; and feeling comfortable discussing RD at work (coded as 1 for yes, 0 otherwise). Explanatory variables included gender; employment grade; years employed; and minority or group status (categorized as racial, ethnic, and/or national minority or group), none, and those who preferred not to answer. We computed frequency distributions of demographic characteristics of survey participants and cross-tabulations of having experienced RD and having witnessed RD by demographic characteristics. Chi-square tests were used to assess differences between groups (p < 0.05).

Where necessary, we translated the qualitative survey data from French into English and subsequently analyzed the data through reflexive thematic analysis, applying an inductive approach and semantic coding using NVIVO Release 1.7.1. Reflexive thematic analysis is a particular type of thematic analysis characterized by its high level of flexibility: this type of analysis does not make use of a code book and allows the researcher to add, remove, and change codes throughout the analysis process. Applying an inductive approach means that the analysis process is approached without any preconceptions, i.e., the themes and sub-themes emerge from the actual data. Semantic coding denotes a particular type of coding in which “codes are identified through the explicit or surface meanings of the data” [28]. Potential hidden meanings and underlying assumptions do not factor into the analysis in this type of coding. We followed the six-phase approach to thematic analysis recommended by Braun and Clarke [29]. This approach involves 1) familiarization with the data; 2) generating initial codes; 3) searching for themes; 4) reviewing potential themes; 5) defining and naming themes; and 6) drafting the results section.

## Results

### Survey participation

A total of 1251 staff, consultants, and interns from a total pool of approximately 8500 eligible employees at the UN in Geneva [27], participated in the survey representing a response rate of 14.7%. Of the 1251 participants, the majority identified as female (59.1%), held professional-level positions (‘Grade P’) (59.5%), and had been employed by the UN for more than ten years (58.9%). The survey offered participants the option to self-identify as a member of one or more minorities or groups. Approximately 36% identified as a member of a racial, ethnic, and/or national minority or group. About half of the participants (53.0%) stated they did not identify as belonging to a minority or group, and some (11.0%) preferred not to answer this question (Table 1).

**Table 1:** Demographic characteristics of survey participants.

	% (N)
***Gender***	
Female	59.07 (739)
Male	39.97 (500)
Other	0.96 (12)
***Employment grade***	
General service staff (G)	31.97 (400)
Professional staff (P)	59.47 (744)
Director (D)	2.88 (36)
Consultant	4.32 (54)
Intern	1.36 (17)
***Years employed by the UN***	
1–5	22.86 (286)
6–10	18.23 (228)
11–15	20.86 (261)
16–20	16.63 (208)
21+	21.42 (268)
***Minority or group belonging (self-identification)***	
Minority or group (racial, ethnic, and/or national minority or group)	35.97 (450)
None	53.00 (663)
Prefer not to answer	11.03 (138)
**Total**	**100 (1251)**

### Racial discrimination in the workplace

We present the results on RD in the workplace in three sub-sections: First, we introduce the findings on personal experience and/or witnessing of RD; second, we report on reasons for and manifestations of RD; and third, we present participants’ responses to RD.

#### Personal experience and/or witnessing of racial discrimination

About one third of the survey participants reported having experienced RD (34.4%) or having witnessed (34.3%) a colleague(s) being racially discriminated against in the workplace (Table 2). People across gender identities reported similar rates of personal experience (33.2% for women, 36.0% for men, 41.8% for other) and witnessed incidents (34.9% for women, 33.5% for men, 30% for other). Gender and employment grade did not show significant associations with personal experience or witnessed incidents of RD (p > 0.05). However, years employed by the UN and personal experience of RD (χ^2^ = 16.62, p < 0.001) as well as witnessing incidents of RD (χ^2^ = 23.56, p < 0.001) were statistically significant. Specifically, participants with one to five years of UN employment reported significantly lower rates of personal experience (16.3%) and witnessed incidents of RD (23.4%) compared to participants with longer tenure (ranging from 32–42%). Moreover, minority or group belonging showed significant associations with personal experience (χ^2^ = 294.79, p < 0.001) and witnessed incidents of RD (χ^2^ = 112.65, p < 0.001). Participants identifying as belonging to a racial, ethnic, and/or national minority or group reported higher rates of personal experience (62.9%) and witnessed incidents of RD (53.2%) compared to those who did not identify as such (13.4% and 21.6%). Our results also show an association between having experienced and having witnessed RD (χ^2^ = 321.39, p < 0.001). Participants who reported having experienced RD had a higher proportion (70.9%) of witnessing RD, whereas those who had not experienced RD had a lower proportion (17.6%) of witnessing such incidents.

**Table 2:** Reported experience and/or witnessing of racial discrimination by demographic characteristics.

	Experienced RD			Witnessed RD			
Explanatory variable	Yes	No	Chi^2^	Yes	No		Chi^2^	
	% (N)	% (N)		% (N)	% (N)			
***Gender***								
Female	33.15 (245)	66.85 (494)	1.36	34.86 (244)	65.14 (456)		0.32	
Male	36.00 (180)	64.00 (320)		33.47 (160)	66.53 (318)			
Other	41.86 (5)	58.33 (7)		30.00 (3)	70.00 (7)			
***Employment grade***								
General service staff (G)	32.5 (130)	67.50 (270)	7.17	31.15 (119)	68.85 (263)		7.18	
Professional staff (P)	34.95 (260)	65.05 (484)		36.17 (255)	63.83 (450)			
Director (D)	44.44 (16)	55.56 (20)		42.42 (14)	57.58 (19)			
Consultant	40.74 (22)	59.26 (32)		32.69 (17)	67.31 (35)			
Intern	11.76(2)	88.24(15)		12.50 (2)	87.50(14)			
***Years employed by UN***								
1–5	16.28 (70)	75.52 (216)	16.62***	23.42 (63)	76.58 (206)		23.56***	
6–10	36.40 (83)	63.60 (145)		37.61 (82)	62.39 (136)			
11–15	39.08 (102)	60.92 (159)		42.40 (106)	57.60 (144)			
16–20	36.54 (76)	63.46 (132)		31.98 (63)	68.02 (134)			
21+	36.94 (99)	63.06 (169)		36.61 (93)	63.39 (161)			
***Minority or group belonging***								
Minority or group	62.89 (283)	37.11 (167)	294.79***	53.21 (224)	46.79 (197)		112.65***	
None	13.42 (89)	86.58 (574)		21.60 (138)	78.40 (501)			
Prefer not to answer	42.03 (58)	57.97 (80)		35.16 (45)	64.84 (83)			
***Experienced RD***						
No	-	-		17.63 (144)	82.37 (673)		321.39***	
Yes	-	-		70.89 (263)	29.11 (108)			
**Total**	**34.37 (430)**	**65.63 (821)**		**34.26 (407)**	**65.74 (781)**			

*** Significance level of explanatory variables at 1%.

#### Reported reasons for and manifestations of racial discrimination

Out of the 385 participants who answered the question on which basis they were racially discriminated, most reported national origin as the basis (61.8%) for the RD they had experienced. Other reasons participants mentioned include race (43.1%), color (37.1%), ethnic origin (21.3%), and descent (13.0%). With regards to how the RD manifested, most participants reported that the incident(s) affected their opportunities for career advancement (66.2%) and having been excluded from work events (37.9%). Furthermore, about one quarter of the participants reported that the RD manifested in verbal abuse (26.8%), and/or having been falsely accused or criticized for wrongdoing (25.7%). A total of 382 participants provided details on which basis their colleague(s) was (were) racially discriminated against. National origin was the most cited basis for these incident(s) (61.0%), followed by race (55.8%), color (53.4%), ethnic origin (24.1%), and descent (16.8%). Regarding how the colleague(s) was (were) racially discriminated against, most participants indicated barriers to career advancement opportunities (51.1%), and more than one third cited exclusion from work events (35.9%) and verbal abuse (34.6%).

#### Responses to racial discrimination

Most participants indicated that they felt comfortable (26.8%) or very comfortable (20.6%) discussing RD in the workplace. Fewer participants felt uncomfortable (13.7%) or very uncomfortable (11.6%) and some participants reported they felt neither comfortable nor uncomfortable (23.7%) or responded with the option ‘I don’t know’ (3.6%). Minority or group belonging demonstrated notable associations with feeling comfortable with discussing RD in the workplace (χ^2^ = 48.34, p < 0.001) (Table 3). Individuals who identified as belonging to an ethnic, racial and/or national minority or groups had the lowest proportion of employees feeling comfortable discussing RD (33.85%) compared to those who preferred not to answer (40.7%) or who identified with none of the minorities or groups (58.6%).

**Table 3:** Proportion of respondents comfortable with discussing racial discrimination.

Explanatory variable	Feeling comfortable to discuss RD		
	Yes	No	
	% (N)	% (N)	Chi^2^
***Gender***			
Female	45.31 (232)	54.69 (280)	2.97
Male	50.28 (178)	49.72 (176)	
Other	66.67 (4)	33.33 (2)	
***Employment grade***			
General service staff (G)	46.44 (124)	55.56 (143)	3.21
Professional staff (P)	47.62 (250)	52.38 (275)	
Director (D)	64.00 (16)	36.00 (9)	
Consultant	44.19 (19)	55.81 (24)	
Intern	46.62 (5)	58.33 (7)	
***Years employed by the UN***			
1–5	50.48 (106)	49.52 (104)	5.80
6–10	47.83 (77)	52.17 (84)	
11–15	42.93 (85)	57.07 (113)	
16–20	54.26 (70)	45.74 (59)	
21+	43.68 (76)	56.32 (98)	
***Minority or group belonging***			
Minority or group	33.85 (110)	66.15 (215)	48.34 ***
None	58.55 (267)	41.45 (189)	
Prefer not to answer	40.66 (37)	59.34 (54)	
**Total**	**47.48 (414)**	**52.52 (458)**	

***Significance level of explanatory variables at 1%.

More than half (57.4%) of the 385 participants who answered the question about actions taken after having experienced RD directly noted they did not take any action. Most indicated a lack of trust in the organization’s recourse mechanism (67.3%) and fear of retaliation from the person(s) involved (55.5%) as reasons for their inaction. Out of those who did take some type of action, many (42.1%) spoke to their colleagues about what they had experienced, few (16.4%) reported the discrimination to their supervisor, and only a small number (1.8%) filed an official complaint. A total of 104 participants provided details on the results of their action. Most (34.6%) stated it did not lead to an end of the behavior of the person(s) involved; others noted that the action made the overall workplace situation worse (17.3%), or that it made the behavior of the person involved worse (12.5%). Only few stated that the action put an end to the behavior by the person(s) involved (12.5%), and about a quarter of those answering this question (23.1%) indicated the category ‘other’.

Participants were also asked what action they took after having witnessed incidents of RD. More than half of the 382 participants who answered this question stated they spoke to their colleague(s) about it (53.4%), while more than one third (36.7%) took no action. The most cited reasons for their inaction were lack of trust in the organization’s recourse mechanisms (77.0%), fear of retaliation from the person(s) involved (44.6%), and fear of retaliation from other colleagues (32.4%). Eighty-four participants provided details on the results of their report/complaint after having witnessed RD. Most stated the action did not lead to an end of the behavior of the persons involved (46.4%), while only few noted that the action put an end to the behavior by the person(s) involved (11.9%).

### Results on how to address racial discrimination in the workplace

**Fig 1:**
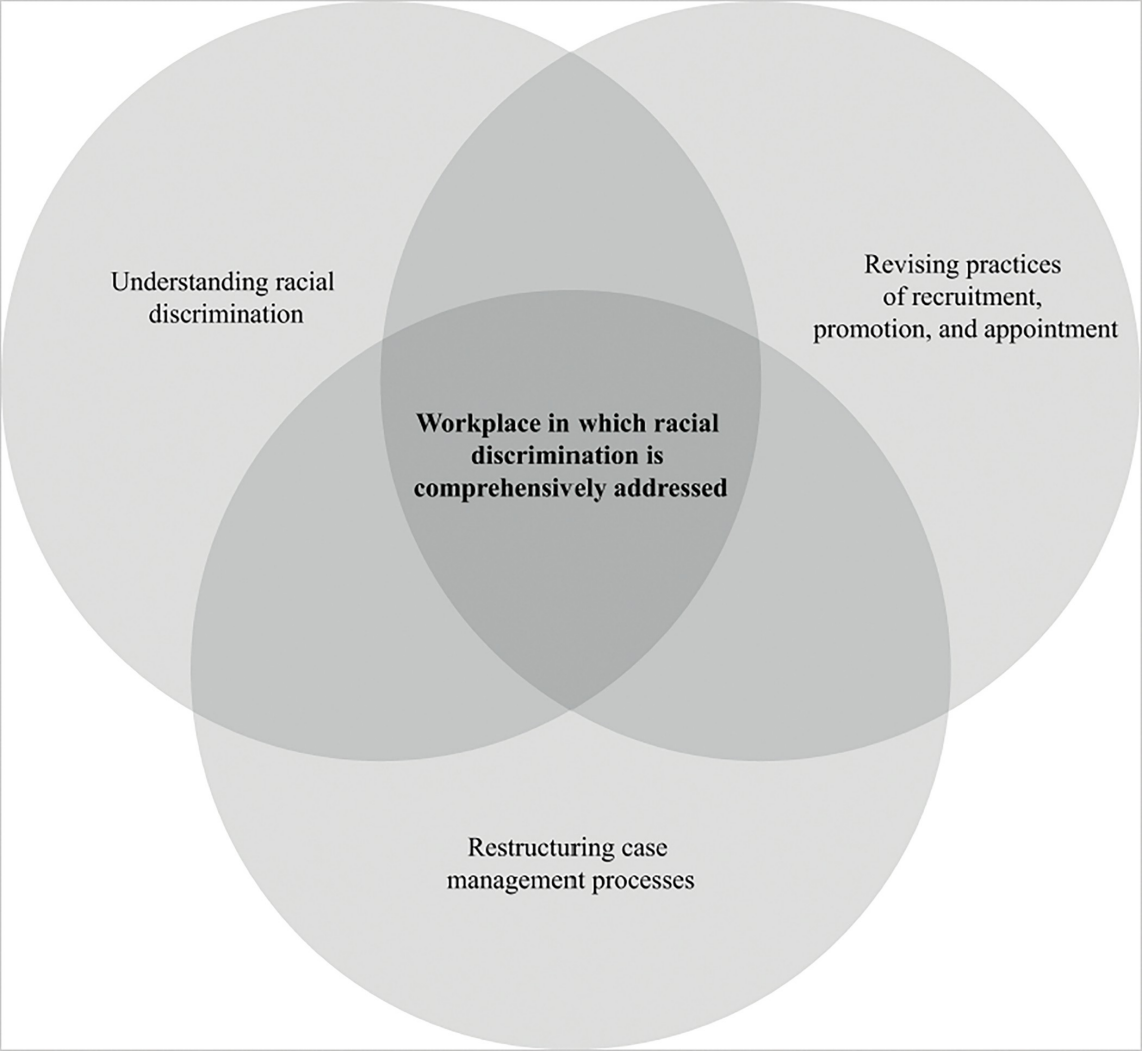
Addressing racial discrimination in the workplace.

A total of 540 participants submitted answers to the open-ended question ‘What measures do you think the United Nations should take to address racial discrimination in the workplace?’ Most of the submitted statements were short, consisting of a few words or sentences only. The thematic analysis of these data rendered three overarching themes: First, understanding RD; second, revising practices of recruitment, promotion, and appointment; and third, restructuring case management processes (Fig 1). We present these themes and their corresponding sub-themes below and contextualize the quoted responses by adding participants’ demographic data on gender, employment category, and minority or group status.

#### Theme 1: Understanding racial discrimination

This theme consists of five sub-themes: a. Acknowledge the issue; b. conduct research; c. raise awareness; d. conduct training; and e. facilitate exchange.

***a*. *Acknowledge the issue*.** Participants highlighted the need for the UN to acknowledge that RD and/or certain forms of racism exist within the organization. While most of the respective statements were vague, a few participants provided detailed suggestions: “We need to understand that racism is embedded in structures, that it is part of engrained power dynamics within our organization, and that we are not immune. Acknowledging that we have a problem is the first step” (female consultant, not belonging to any minority or group). Furthermore, some participants emphasized the need to acknowledge and address prevailing privileges. As one participant put it: “Acknowledge that the world is diverse, and that the ways of the western world [are] not the only right way. We can all co-exist in diversity by respecting different methods and viewpoints, and different beliefs, colour, cultures, language, approaches” (female P-staff, member of a racial, ethnic, and national minority or group). That RD can be a complicated subject matter that requires careful studying was also mentioned:

“Realize that it’s [RD] contextual. I can feel that I am part of a minority group working in Japan or Nigeria or Samoa but not in Belgium or [the] Philippines or Bhutan. Minority is relative to the prevailing majority. Just being black or white or asian [sic] doesn’t make you subject to discrimination if you’re working in a primarily black or white or asian [sic] context” (male P-staff, preferred not to answer question on minority / group status).

***b*. *Conduct research*.** To better understand RD within the UN, participants suggested conducting further research on the topic. One participant was very explicit in this regard:

“Examine what prejudiced narratives we are perpetrating about Africa through our communications, meetings and publications: [First,] examine employment contracts administered, e.g., pay scales, lengths of contract, appropriate immigration support for Africans. [Second,] examine access to other services, i.e., banking, housing etc. While services are not withheld at the UN, external parties do discriminate against minorities, making it harder for us to settle into our position at work” (female consultant, member of a racial minority or group).

Other suggestions regarding research on RD in the workplace included running anonymous surveys more regularly, conducting exit interviews or surveys with staff leaving teams or the UN system, and targeted research on “unconscious bias regarding expertise and leadership” (female P-staff, member of a racial and ethnic minority or group) as well as on “interpersonal and institutional biases towards racial minorities” (female consultant, member of a racial minority or group).

***c*. *Raise awareness*.** Participants also suggested to “give even more visibility to these important issues” (male G-staff, not belonging to any minority or group) and to raise awareness but mostly remained vague regarding whose awareness should be raised on what and how. Some wrote about the need to issue awareness programs, meetings, or a full campaign, including a “shock campaign (photos and testimonies)” (female G-staff, not belonging to any minority or group). Furthermore, one participant noted that showing short videos could help. Only one participant provided more specific details when requesting to “raise awareness on different types of national, ethnic and national/international staff discrimination that exist in different regional or national contexts” (female P-staff, not belonging to any minority or group).

***d*. *Conduct training*.** Conducting mandatory training was one of the most suggested interventions by participants, and many highlighted the strong need for the UN to invest in “educational actions” (male G-staff, not belonging to any minority or group). A significant proportion of these participants suggested the training should specifically target those in leadership or management positions, i.e., “people in positions of power” (female P-staff, not belonging to any minority or group). As one participant put it: “I think P4 [level] onwards need(s) to be given more training on the subtle ways discrimination is practiced and how unconscious biases affect this” (female P-staff, member of a national minority or group). Other specific target groups that were mentioned in the context of needing training include “Western colleagues” (female P-staff, member of an ethnic minority or group) and “hiring managers and HR [Human Resources]” (female P-staff, not belonging to any minority or group).

With regards to the content of the training, some participants brought forward precise suggestions. These included requests for training on definitions of important terminology including the following: micro-insults, micro-invalidations, micro-assaults, and gaslighting; unconscious biases and organizational redress mechanisms; cultural biases; decolonization; and non-racist behavior. In terms of format, one participant stated the need for “(m)ore training, but not only online training. This is a topic where person to person interaction can probably be more impactful” (female P-staff, member of a racial minority or group). Similarly, referring to previous experiences, another participant wrote: “The anti-discrimination measures I’ve taken part in have mostly been online and through training which did not involve peer-to-peer interactions which I think would have a much greater impact on empowering people to better understand, resist and overcome it” (male P-staff, not belonging to any minority or group). Furthermore, a few others noted the power of video testimonials and “(r)ole-playing workshops (putting oneself in the shoes of those who are discriminated against) to change the way we look at things and people” (male G-staff, not belonging to any minority or group).

***e*. *Facilitate exchange*.** Another suggested measure to be facilitated by the UN was exchange between members of the workforce. Indeed, numerous participants voiced their desire to talk openly and honestly with colleagues about the issues of racism and discrimination more broadly, including prejudices and the use of language, thereby requesting the UN to encourage dialogue and “normalize conversations around discrimination” (male P-staff, member of a racial minority or group). For some, this implied “creat(ing) [a] safe space for staff to be able to discuss racism and share their stories” (female P-staff, member of a racial minority or group). One participant also suggested to “organize inter-racial / ethnic minorities focus groups to talk about discrimination” (female P-staff, not belonging to any minority or group).

#### Theme 2: Revising practices of recruitment, promotion, and appointment

This theme consisted of the following four sub-themes: a. Change the recruitment process; b. enhance diversity and affirmative action; c. reconsider performance reviews; and d. appoint special advisers.

***a*. *Change the recruitment process*.** A large proportion of the suggestions shared by participants focused on the need to change organizations’ recruitment processes. Specifically, many participants requested recruitments to be fair, transparent, and impartial, with the criteria candidates are evaluated against being focused on competence. However, the reasoning behind this focus on competence differed between participants. For example, one staff stated that “the best person should be recruited, not based on race, sex or geographic reasons” (male P-staff, not belonging to any minority or group). Similarly, a few others recommended to “(r)eturn to selection and promotion decisions in accordance with the UN Charter, based on merits and performance, not nationality and gender only” (male P-staff, not belonging to any minority or group). Another participant, however, emphasized the need to “ensure that people with ‘local knowledge’ due to their nationality, or with ‘language skills’, are not under-valued or placed into junior positions that they accept but are not commensurate with their experience” (female P-staff, member of a racial and ethnic minority or group). Similarly, one participant wrote:

“Favoritism based on nationality/passport is still very noticeable in the UN and certain managers prefer to hire P-staff [professional-level staff] from certain countries (North America and Europe) whenever given the opportunity, even if there are more suitable candidates from Africa, Asia and/or [the] post-Soviet region for example [who] keep proving every day that they are also experienced, capable and worthy of equal chances for promotion” (female G-staff, member of an ethnic and national minority or group).

One participant explicitly cautioned against “the commendable push for geographic diversity at all levels in the system.” According to him, this “can translate into often well intended but possibly discriminatory practices of putting diversity above competence in recruiting or promoting personnel” (male consultant, preferred not to answer question on minority / group status).

***b*. *Enhance diversity and affirmative action*.** Strongly related to the previous sub-theme are the many submissions that are directly centered on diversity and affirmative action as tool towards achieving greater diversity. While some statements, for example to “build a diversified workplace” (female consultant, member of a racial, ethnic, and national minority or group) and to “promote diversity” (male P-staff, preferred not to answer question on minority / group status) were vague, other participants specified a need for greater diversity of staff with regards to their nationality, region, race, or ethnicity. One participant explicitly wrote about the issue of geographic versus racial diversity:

“The UN does not seem to take into account in any way the ethnic and racial composition of its staff—unlike any other organisation in the world. Instead, there is a focus on geographic representation as a replacement. This however is an insufficient replacement which is not fit for purpose” (male P-staff, member of an ethnic minority or group).

Further, some participants highlighted the need for diversity at higher levels. As expressed by one female staff member: “Institute leadership that is not exclusively from Germany, France, UK, USA, the Netherlands (the West)” (female P-staff, preferred not to answer question on minority / group status). Another participant echoed this request: “Give more space to people of color, senior management is often composed by men, white, from developed countries” (female consultant, member of a national minority or group).

Affirmative actions, such as offering “more job opportunities for qualified minorities” (female G-staff, member of a racial minority or group) and “actively promoting and supporting the employment and career development of people belonging to a racial or ethnic minority especially coming from low- and middle-income countries” (female P-staff, member of a national minority or group) were mentioned as measures to achieve greater diversity. With regards to achieving inclusion at higher levels, one participant recommended to specifically “promote individuals from underrepresented countries to decision making positions” (male P-staff, preferred not to answer question on minority / group status).

To implement affirmative action and achieve greater diversity, participants mentioned the need for the collection of data disaggregated by gender, nationality, self-reported minority status, race, and/or ethnicity. One participant requested the circulation of such data: “There should be a regular publication of statistics relating to each and every recruitment and promotion broken down by nationality and race, showing the details of those who were in the long list, short list, interviewed and selected” (female P-staff, member of a racial minority or group).

***c*. *Reconsider performance reviews*.** Performance reviews were the subject of discussion in that some participants requested to include RD into the review system, especially with regards to those in managerial positions: “Include diversity indicators (both race and gender) as part of management evaluations” (female P-staff, member of a racial and ethnic minority or group), wrote one participant. Another stated that “the evaluation of senior manager’s performance should include questionnaires about RD. The assessment should be limited to directly supervised staff but encompasses sampling from all the team working under their authorities” (male P-staff, member of a national minority or group). At the same time though, a few participants explicitly requested to “clearly separate discussions about performance evaluation from racial discrimination”, noting it was not “(…) useful to collapse everything in one. Racism can exist in these areas but also outside. Equally performance issues can exist without being tied to race” (female P-staff, not belonging to any minority or group).

***d*. *Appoint special advisers*.** Given the challenges with addressing RD, participants suggested the appointment of one or more special advisers or dedicated senior officers well-positioned to guide respective processes. Suggestions towards this end included that the adviser(s) should work “independently and report directly to the SG [Secretary General]” (male P-staff, member of a racial minority or group), to open a specific section dealing with the issue of RD within the Ombudsman’s Office, and to create an “anti-racism office with focal points in all duty stations” (female P-staff, member of a racial minority or group).

#### Theme 3: Restructuring case management processes

Four sub-themes make up this theme. These are: a. Develop policies and plans; b. establish reporting and investigation mechanisms; c. move from words to action; and d. impose strict measures.

***a*. *Develop policies and plans*.** Developing written documents, especially policies and plans, that guide the UN’s work on RD was important to participants. For example, one participant suggested to prepare an “anti-racist action plan” (male consultant, not belonging to any minority or group) and another one requested the preparation of a “non-discrimination policy” (male P-staff, not belonging to any minority or group). These documents should be detailed, outlining all steps to be taken by the organization to address RD.

***b*. *Establish reporting and investigation mechanisms*.** Establishing a reporting mechanism was considered a crucial step in addressing RD. To be useful, this mechanism should be transparent and fair, known to colleagues, and easy to use. The concepts of anonymity and confidentiality were also mentioned as important as well as the need to “make people feel comfortable reporting such discrimination in the workplace” (male P-staff, member of an ethnic and national minority or group), including protection of their jobs given that “many people do not report due to contract insecurity” (male consultant, not belonging to any minority or group). In line with these remarks, one participant elaborated on the perceived challenges with reporting at the time and suggested an alternative: “Have a hotline that is open and ensure that retaliation is addressed. Most of the time complaints on racism are underplayed and there is no concrete evidence and people are made to feel that they are destroying the work environment by reporting” (female P-staff, member of a racial, ethnic, and national minority or group). Another suggestion shared by participants was to report to an external party outside the UN system.

To address RD and handle the reported cases, participants recommended to issue more regular investigations. These should be impartial, unannounced, and conducted by external evaluators. One participant shared detailed views on the process of investigations, suggesting that employees

“should be able to file a complaint on these so serious grounds directly against another employee to an independent labor tribunal, without having to undergo a complicated (politically) process/procedure of management decision evaluation (as in some cases managers are accomplices…), and without fear of retaliations” (female P-staff, member of a racial and ethnic minority or group).

***c*. *Move from words to action*.** Participants highlighted the urgent need to move from words to action. For example, one participant noted “we talk a lot and do nothing when it comes to taking action. We need action. We need to learn to respect one another” (female P-staff, member of a racial minority or group). Others confirmed the importance of “find(ing) a channel for the reports to actually be acted upon for better behavior” (female G-staff, not belonging to any minority or group) and to “stop paying lip service, [and] engage in action” (male P-staff, preferred not to answer question on minority / group status). In this context participants also wrote that it was important to enforce existing policies and measures, to prevent the loss of trust from staff in their employer:

“Most importantly, the organization must enforce those measures so that words be followed by action. I have personally lost faith in the UN because I can see that perpetrators of discrimination, harassment and bullying are not punished for their acts, but rather promoted and protected” (female P-staff, member of a racial and national minority or group).

***d*. *Impose strict measures*.** Participants shared various strict measures to be implemented to address RD in the workplace. Holding colleagues accountable for their behavior was one prominent suggestion in this context. This was especially requested for managers, including with regards to diverse tasks such as “develop[ing] actionable guidelines” (female P-staff, member of a racial and national minority or group); “how they assign work to staff they supervise” (female P-staff, member of a racial minority or group); the “lack of progress in diversity of staff, including staff on temporary contracts” (male P-staff, member of a racial and ethnic minority or group); and “the performance at the level of their team” (male P-staff, member of a national minority or group). One participant also noted the importance to “apply inside the organization, what it preaches outside to member States” (female P-staff, not belonging to any minority or group), while another one pointed out that after having ensured that “the accusation of wrongdoing is founded” the perpetrator must be held accountable, including through “disciplinary, immediate and concrete measures” (male P-staff, member of a racial minority or group).

This need for disciplinary action was echoed by others and included multiple calls for a zero-tolerance approach and harsh penalties, as well as “strong credible sanctions (dismissal, instead of sinking to the top type of practices, so often the sad reality in this house)” (female P-staff, not belonging to any minority or group). Participants also suggested to discharge staff who commit RD. In this context, some touched upon the issue of impunity. As one participant put it: “Discipline managers especially at the mid-level who perpetuate bad practices (…) to get things done and then are praised for this instead of disciplined for creating a toxic, and extremely negative working environment” (female P-staff, member of a racial and ethnic minority or group).

References to the successful gender work undertaken within the UN were also common, including the suggestion to use this work as guidance for addressing RD: “Similar to the gender balance initiative, the Organization should take race into consideration for racial equality at all levels” (male P-staff, member of a racial and ethnic minority or group). Further, one participant mentioned the need to consider how gender and race intersect in the context of discrimination.

## Discussion

The results we presented in this article indicate that RD is a significant issue within UN offices in Geneva, home to the second largest UN center globally. They can be used to justify and inform the scaling up of existing anti-racism strategies and the creation of internal plans tailored to this specific setting. Our findings are both expected and surprising:

First, reported rates of personally experienced RD (about one third of participants), the basis for this discrimination (mainly national origin, followed by discrimination based on race and color), and inaction as the main response due to a lack of trust in the organization’s recourse mechanism and fear of retaliation, are consistent with the findings from other UN surveys launched elsewhere at a similar point in time [13]. Reported rates of personal experience and witnessed incidents of RD were significantly higher for those with a longer tenure in the organization. This finding is intuitive, given that more years in the workplace typically come with more opportunities for discrimination. As expected, participants who identified as belonging to a racial, ethnic, and/or national minority or group reported higher rates of experienced and witnessed incidents of RD compared to those who did not identify as such. We also found that participants who reported having experienced RD had a higher proportion of witnessing RD than those who did not personally experience this form of discrimination. This can likely be explained through a high level of sensitivity and awareness of the issue among those who reported having personally experienced RD. Regarding the interpretation of these and further results, it is important to note that they are based on the reporting of subjective experiences. While this is common for surveys on racial and other forms of discrimination [30], these findings do not, in the absence of professional evaluations and investigations of the respective incidents, allow for the drawing of conclusions on the prevalence of legally confirmed RD within the UN. However, it is accepted within the literature to characterize the reported experiences as a valid form of stress, even in the absence of verification [31].

Second, while it is alarming that only a few participants reported having personally experienced and/or witnessed cases of RD through official channels, this finding is not surprising. Underreporting of incidents of RD remains common in the world of work [32,33], and various studies with different occupational groups across countries rendered similar results [34,35]. Encouraging is that out of those participants who did take some type of action after having experienced and/or witnessed RD, many chose to speak to their colleagues about these incidents. This suggests members of the workforce possess the required level of empathy and willingness to provide support, indicating the existence of positive interpersonal relationships at work.

Third, considering the strong hesitation to officially report cases of perceived RD, it is somewhat surprising that almost half of those who answered the question stated feeling comfortable with discussing issues of RD at work. This is even more the case given that many people, and especially white people, typically perceive discussions about race and racism as challenging [36]. One explanation for this paradox might be that, while open conversations about systemic racism have become culturally acceptable in the workplace and other spheres of social organization, especially following the murder of George Floyd, conversions about specific incidents may remain challenging due to perceived risks to status and wellbeing [37].

Fourth, the detailed analysis of suggestions of how to address RD resulted in a variety of different measures, including investments in initiatives that lead to a better understanding of RD (and racism more broadly), and significant structural changes. These measures are, by and large, in line with the comprehensive set of actions recommended in the Strategic Action Plan released in 2021 by the Secretary General’s Task Force on Addressing Racism and Promoting Dignity for All in the United Nations Secretariat. Specifically, this plan is aimed at addressing structural, institutional, and personal racism in four areas, i.e., organizational culture; operations and management practices; systems, including structures and policies; and internal mechanisms of accountability and safe complaints handling [13]. However, our analysis also highlights the need for the restructuring of case management processes, including the suggestion for employees to report perceived cases of RD to an external entity outside the UN system, and to hire external investigators to handle these. While the Strategic Action Plan covers recommendations centered on reporting and investigations, including “an external independent sample review of past complaints of racism” [13], it does not provide for the option to either fully outsource case management processes, or to do so on a case by case basis. Hiring an external investigator can be interpreted as a signal that organizations take the situation in question seriously, thereby enhancing employees’ confidence and trust in the organization [38]. Further, external investigators are typically perceived as more neutral and are thus generally more successful in getting personnel to open-up without fearing retaliation [39]. Given the strong hesitation to formally report RD that our analysis revealed, it remains to be seen if UN employees will feel more comfortable with reporting experienced and/or witnessed incidents than they have in the past if reporting and investigations continue to be exclusively handled in-house.

Finally, our results revealed a concern that a strong emphasis on diversifying the workforce might come at the cost of competence and performance. This raises the question of how competence and performance are being implicitly and explicitly defined within the UN system and by whom. As Bian [14] observed in the context of the humanitarian workplace across organizations:

“Although expertise and competency are not directly visible, it is often associated with visible characteristics. In today’s humanitarian space, one’s whiteness can be a very covert yet common prerequisite for professional recognition as ‘ideas about black inferiority precede professional encounters.’”

Given this propensity for RD, affirmative action strategies may be implemented to counter the structural disadvantages experienced by Black and other racialized groups and promote professional advancement. Yet, such strategies continue to come under scrutiny [40] despite significant agreement in the literature that workplace diversity can increase productivity and enhances an organization’s image, reputation, and economic position. This frequently cited rationale for valuing diversity in the workplace is called the ‘business case’ [41]. However, for the mentioned benefits to manifest, diversity management, understood “as a process intended to create and maintain a positive work environment where the similarities and differences of individuals are valued, so that all can reach their potential and maximize their contributions to an organization’s strategic goals and objectives” [42] is required. Otherwise, diversity might indeed bear the risk to cause misunderstandings, suspicion, and even conflict among employees, thereby leading to adverse outcomes, such as low morale, absenteeism, and reduced work quality [43].

## Limitations

The survey on RD has four limitations: First, it was limited in scope in that it assessed RD only but did not elicit opinions and experiences regarding racism in the workplace more broadly, including the impacts thereof on health and well-being. The survey also did not investigate intersectionality, i.e., the multiplicative effects of interrelated identity-shaping factors such as race in connection with gender identity, sexual orientation, ability, or age for example [44,45]. Second, as noted by few participants, some questions were perceived as challenging to answer in the absence of definitions or explanations of relevant terminology (e.g., the differences between national origin/race/color/ethnic origin/descent as basis for RD). Third, the response rate of 14.7% is low for employee surveys [46], but in line with that of another online survey with staff from the UN and non-governmental organizations on sensitive workplace issues [10]. Fourth, it might be that certain employees, e.g., those who have experienced or witnessed RD, felt more drawn to complete the survey than others, resulting in self-selection bias.

## Conclusion and recommendations

Amid global calls for racial justice, anti-racism must be at the core of humanitarian and development work [47,48]. However, a rigorous examination of how humanitarian / development organizations uphold the values of anti-racism must be directed both outwards and inwards. This analysis of survey data demonstrated that RD poses a significant issue within the UN offices in Geneva with almost two-thirds of participants indicating that their direct or indirect experience with RD was related to discrimination based on national origin. Reported rates of personal experience and witnessed incidents of RD were significantly higher for those with a longer tenure in the organization and affected respondents across gender identity. Reported incidents of RD took the form of verbal abuse, being falsely accused or criticized for wrongdoing, barriers to career advancement opportunities, and exclusion from work events.

Given the significant associations between RD, racism more broadly and health, especially mental health, that have been documented in previous studies [3], future research should examine the relationship between reports of RD, racism and incidents of sickness, presenteeism, and absenteeism amongst employees [7,8]. This has been vastly neglected in the UN discourse on RD and racism in the workplace. Correlating subjective reports of RD and racism with documented incidents of RD and racism within the workplace and relevant data from exit interviews could provide further support for the formulation of systemic strategies to address RD and racism within the UN. We recommend that the UN and other humanitarian / development organizations collaborate closely with researchers in these endeavors to ensure the rigorous collection and analysis of encompassing data relevant to the topic.

Participants presented a variety of tailored measures to address RD within the organization, including educational initiatives and significant structural changes. The existence of confidants within the organization, the openness of survey participants to talk about RD in the workplace, and their requests for the UN to facilitate exchanges between employees on this topic suggest a willingness to work towards needed change. We recommend that this momentum be utilized, for example in the context of ally development as one tool for achieving social justice in the workplace [49]. However, it must be noted that strategies that place undue stress on racialized groups or place them at higher risk of retaliation must be avoided; suitable and skilled leadership is needed to ensure the success of respective steps [9,50]. Thus, we also recommend additional research on conducive approaches to dismantling structural discrimination alongside effective methods of reporting and responding to individual incidents.

In sum, acknowledging, understanding, and challenging RD and racism and its underlying ideologies in central to public health including the health of those who work within humanitarian and development organizations. Consistent with the vision of the Task Force on Addressing Racism and Promoting Dignity for All in the United Nations Secretariat [13], ensuring that “everyone has an equal opportunity to participate in the work of the Organization and is treated with respect and dignity” will require committed leadership and the participation and vigilance of all involved.
